# Tumour Behaviour of Low-Grade Papillary Urothelial Carcinoma: A Single-Centre Retrospective Study

**DOI:** 10.7759/cureus.16012

**Published:** 2021-06-29

**Authors:** Satya Dutta, Biswajit Dey, Vandana Raphael, Yookarin Khonglah, Jaya Mishra, Evarisalin Marbaniang, Pranjal Kalita, Stephen Sailo

**Affiliations:** 1 Pathology, North Eastern Indira Gandhi Regional Institute of Health and Medical Sciences (NEIGRIHMS), Shillong, IND; 2 Urology, North Eastern Indira Gandhi Regional Institute of Health and Medical Sciences (NEIGRIHMS), Shillong, IND

**Keywords:** urothelial carcinoma, recurrence, grade

## Abstract

Background and objective

Carcinoma of the urinary bladder is the most common urological cancer, and it accounts for 3.9% of all cancer cases in men. Patients with the subset of noninvasive low-grade papillary urothelial carcinoma (LG-UrCa) are at higher risk for tumour recurrence. In this study, we aimed to analyse the histopathological features of LG-UrCa and to correlate those with recurrence potential as well as disease stage and grade progression.

Materials and methods

We conducted a retrospective study from January 2016 to December 2018. All cases with presenting biopsy initially reported as LG-UrCa were included in the study. All cases with initial biopsy reported as high-grade papillary urothelial carcinoma (HG-UrCa) were excluded from the study. We used the 2016 World Health Organization/International Society of Urological Pathology (WHO/ISUP) guidelines for the classification of papillary urothelial neoplasm.

Results

A total of 48 initially diagnosed cases of LG-UrCa were identified. Two out of 48 cases were reclassified as high-grade urothelial carcinoma and were excluded from the study. The mean age of patients at presentation was 56.7 years. The mean duration of follow-up was 19.8 months. The mean size of initial tumours was 3.4 cm. Tumour recurrence was encountered in 14 (30.4%) of 46 patients. Out of the four patients who had high-grade progression (8.7%), two also developed TNM stage progression. These two patients eventually underwent radical cystectomy. Patients with larger initial tumour sizes were found to have an increased tumour recurrence rate (p=0.009). Patients with multiple lesions at initial diagnosis had a significantly higher tumour recurrence rate than those with a single tumour (p=0.02). There was no significant difference with regard to intravesical Bacillus Calmette-Guérin (BCG) and tumour recurrence (p=0.065). None of the clinicopathological parameters were significantly associated with the grade and/or stage progression.

Conclusion

Based on our findings, patients with larger initial tumour size and tumour multiplicity at presentation had an increased tumour recurrence rate.

## Introduction

Urothelial carcinoma consists of carcinoma of the urinary bladder, ureters, and renal pelvis [1]. Among these, urinary bladder cancer (BCa) is the most common urological malignancy, and it accounts for 3.9% of all cancer cases in men [1]. The outcomes of the noninvasive papillary tumour, i.e., tumour recurrence and tumour progression, largely depend on the tumour pathological grade [2]. Several classification systems have been proposed for the pathological grading of noninvasive papillary lesions of the urinary bladder [3]. In 2004, the World Health Organization (WHO) adopted the International Society of Urological Pathology (ISUP) classification of flat and papillary lesions of the urothelium, and the WHO/ISUP system was instituted [4]. The recent 2016 WHO Blue Book continues to endorse the ISUP classification [5]. According to this, urothelial lesions are categorised into infiltrating urothelial carcinoma and noninvasive urothelial neoplasm [6]. Noninvasive urothelial neoplasms are further classified into various categories: papilloma, inverted papilloma, papillary urothelial neoplasm of low malignant potential (PUNLMP), low-grade papillary urothelial carcinoma (LG-UrCa), high-grade papillary urothelial carcinoma (HG-UrCa), urothelial carcinoma in situ, and urothelial dysplasia [6]. LG-UrCa is characterised by an overall orderly arrangement of cells with minimal variability of cellular architectures, and lack of significant nuclear atypia and mitotic activity [7]. Patients with LG-UrCa are at risk of tumour recurrence with a few of them developing higher grade disease and stage progression [8].

In the present study, our objective was to evaluate the clinicopathological parameters of recurrence and progression in patients with LG-UrCa. We also aimed to analyse the various outcomes in the patients.

## Materials and methods

This was a retrospective study conducted from January 2016 to December 2018. All the cases of urothelial carcinoma were included in the study and re-evaluated by two uropathologists. All the cases with presenting biopsy initially reported as LG-UrCa were included in the study. All the cases with initial biopsy reported as HG-UrCa were excluded from the study. We used the 2016 WHO/ISUP guidelines for the classification of papillary urothelial neoplasm [5]. Tumour size and multifocality of the tumours were recorded from the cystoscopy reports. The outcomes of the disease and other clinical data were obtained by reviewing our hospital medical records.

Grading of papillary urothelial neoplasm

Low-Grade

Papillary urothelial neoplasm was graded as a low-grade if it was characterised by an overall orderly cellular appearance but with mild variation in architectural and cytological features like uniformly enlarged, hyperchromatic nuclei with fine chromatin and small or inconspicuous nucleoli and occasional mitotic figures.

High-Grade

Papillary urothelial neoplasm was graded as a high-grade if it was characterised by the disorderly appearance of both cytonuclear and architectural disorganisation with moderate to marked cellular pleomorphism, prominent nucleoli, and frequent mitosis. Architecturally, HG-UrCa is associated with fused papillae and an anarchic growth.

Staging of papillary urothelial neoplasm

The staging was done as per the American Joint Committee on Cancer (AJCC) TNM staging system, 8th edition.

Stage 0a

This is early cancer that is only found on the surface of the inner lining of the bladder. Cancer cells are grouped together and can often be easily removed. Cancer has not invaded the muscle or connective tissue of the bladder wall and can be both low- or high-grade. This type of bladder cancer is also called noninvasive papillary urothelial carcinoma (Ta, N0, M0).

Stage 0is

This stage of cancer, also known as a flat tumour or carcinoma in situ (CIS), is found only on the inner lining of the bladder. This is invariably high-grade cancer. It has not grown in toward the hollow part of the bladder, and it has not invaded the muscle or connective tissue of the bladder (Tis, N0, M0).

Stage I

Cancer has grown through the inner lining of the bladder and invaded the lamina propria. However, It has not invaded the bladder wall muscle or lymph nodes, or other organs (T1, N0, M0).

Stage II

Cancer has invaded the thick muscle wall of the bladder. It is also called invasive cancer or muscle-invasive cancer. However, the tumour has not reached the fatty tissue surrounding the bladder or the lymph nodes or other organs (T2, N0, M0).

Stage IIIA

Cancer has grown into the perivesical tissue or has spread to the prostate, uterus, or vagina, but has not spread to the lymph nodes or other organs (T3a, T3b, or T4a; N0; M0), or cancer has spread to a single regional lymph node (T1 to T4a, N1, M0).

Stage IIIB

Cancer has spread to two or more regional lymph nodes or to the common iliac lymph nodes (T1 to T4a, N2 or N3, M0).

Stage IVA

Cancer has spread to the pelvic wall or the abdominal wall but not to other parts of the body (T4b, N0, M0), or cancer has spread to lymph nodes located outside of the pelvis (any T, any N, M1a).

Stage IVB

Cancer has spread to other parts of the body (any T, any N, M1b).

Statistical analysis

For statistical analysis, the chi-square test or Fisher's exact test was performed for categorical variables. Comparison of means between two groups was tested by the independent samples t-test using MedCalc version 20.008 (MedCalc Software, Ostend, Belgium). A p-value of less than 0.05 was considered to be statistically significant.

## Results

A total of 48 cases initially diagnosed as LG-UrCa were re-evaluated. Two uropathologists reviewed these cases, and two cases (4.16%) were reclassified as HG-UrCa, which were excluded from the study. The age of the patients ranged from 30 to 89 years with a mean age at presentation of 56.7 years. There were 38 males (M) and eight females (F) with an M:F ratio of 4.75:1. The follow-up period ranged from two to 60 months with a mean follow-up period of 19.8 months. None of the patients died till the last follow-up.

Of note, 30 (65.2%) out of 46 patients presented with hematuria; 24 patients (52.1%) were either current smokers or had a past history of smoking, whereas three were non-smokers. The smoking history for the rest was unknown. Nineteen patients (41.3%) had a single initial lesion, 27 (58.7%) had multiple lesions at initial transurethral resection (TUR). The mean initial tumour size was 3.4 cm (range: 1-9.3 cm). Repeat urine cytology and cystoscopy were followed at three, six, and 12 months for the first year, every six months for the second year, and annually thereafter. In 11 patients (23.9%), intravesical Bacillus Calmette-Guérin (BCG) was administered (Table 1).

**Table 1:** Patient demographic and tumour characteristics

Variables	Values	
Total number of cases	46	
Males	38	
Females	8	
Mean age at presentation	56.7 years	
Smoking, n (%)		
None	3 (6.5%)	
Unknown	19 (41.3%)	
Previous	3 (6.5%)	
Current	21 (45.7%)	
Hematuria, n (%)		
Present	30 (65.2%)	
Absent	16 (34.8%)	
Mean/median initial size in cm (range)	3.43/3 (1-9.3)	
Number of lesions on initial presentation, n (%)		
Single lesion	19 (41.3%)	
Multiple lesions	27 (58.7%)	
Intravesical Bacillus Calmette-Guérin (BCG) therapy, n (%)		
Given	11 (23.9%)	
Not given	35 (76.1%)	
Recurrence, n (%)		
Present	14 (30.4%)	
Absent	32 (69.6%)	
Grade progression, n (%)		
Present	4 (8.7%)	Out of 4 cases that had grade progression, 2 cases (4.34%) also showed stage progression
Absent	42 (91.3%)	

All the 46 cases were staged at stage 0a. Of these cases, 14 (30.4%) developed one or more episodes of recurrence; 10 (21.7%) out of 46 cases had recurrence as LG-UrCA according to 2016 WHO/ISUP classification and all were staged at stage 0a [5]. Four out of 46 cases (8.7%) showed grade progression, developing HG-UrCa in one or more recurrence episodes, and were staged at stage 0a. Two out of four patients with recurrent HG-UrCa also developed stage progression (stage II) and underwent radical cystectomy (Table 1).

Patients with multiple lesions at initial diagnosis had a significantly higher recurrence rate (85.7%; 12/14) than those with a single tumour (p=0.02) (Table 2). However, the presence of multiple tumours on TUR was not associated with significant grade (p=1.0) or grade/stage progression (p=1.0) (Tables 3, 4). There was a significant difference in mean tumour size between cases with and without recurrences (mean size of 2.93 versus 4.53 cm respectively; p=0.009) (Table 2). However, there was no significant difference between initial tumour size and grade progression (mean size of 3.5 versus 3.75 cm; p=0.8) or grade/stage progression (mean size of 3.5 versus 5 cm; p=0.28) (Tables 3, 4).

**Table 2:** Clinicopathological parameters and tumour recurrence SD: standard deviation

Parameters	No recurrence (n=32)	Recurrence (n=14)	P-value
Mean age in years (±SD)	55.71 (±15.08)	58.92 (±13.08)	0.49
Sex, n			0.40
Male	25	13	
Female	7	1	
Smoking history, n			0.83
None	2	1	
Previous	3	2	
Current	15	4	
Unknown	12	7	
Mean tumour size in cm (±SD)	2.93 (±1.68)	4.53 (±2.14)	0.009
Number of lesions, n			0.02
Single	17	2	
Multiple	15	12	
Intravesical Bacillus Calmette-Guérin (BCG) therapy, n			0.065
Yes	5	6	
No	27	8	

With regard to intravesical BCG therapy, there was no statistically significant difference between the groups with and without recurrence (p=0.065), with and without grade progression (p=1.0), or with and without grade/stage progression (p=0.44) (Tables 2, 3, 4). Although the mean age was higher in patients with recurrence, grade, and stage progression, the differences were not significant with regard to tumour recurrence (mean age of 55.71 versus 58.92 years; p=0.49), with grade progression (mean age of 56.57 versus 59.75 years; p=0.68), or with grade/stage progression (mean age of 56.57 versus 61.5 years; p=0.65) (Tables 2, 3, 4). We found no significant association between patient sex or smoking history and tumour recurrence (Table 2).

**Table 3:** Clinicopathological parameters and grade progression SD: standard deviation

Parameters	No grade/stage progression (n=42)	Grade progression (n=4)	P-value
Mean age in years (±SD)	56.57 (±15.03)	59.75 (±7.63)	0.68
Sex, n			1.0
Male	34	3	
Female	8	1	
Smoking history, n			0.41
None	3	1	
Previous	4	1	
Current	19	0	
Unknown	16	2	
Mean tumour size in cm (±SD)	3.5 (±1.91)	3.75 (±1.7)	0.8
Number of lesions, n			1.0
Single	17	2	
Multiple	25	2	
Intravesical Bacillus Calmette-Guérin (BCG) therapy, n			1.0
Yes	10	1	
No	32	3	

None of the clinicopathological parameters had any significant association with tumour grade or combined grade/stage progression (Tables 3, 4).

**Table 4:** Clinicopathological parameters and grade/stage progression SD: standard deviation

Parameters	No grade/stage progression (n=42)	Grade and stage progression (n=2)	P-value
Mean age in years (±SD)	56.57 (±15.03)	61.5 (±12.02)	0.65
Sex, n			0.37
Male	34	1	
Female	8	1	
Smoking history, n			0.82
None	3	0	
Previous	4	1	
Current	19	0	
Unknown	16	1	
Mean tumour size in cm (±SD)	3.5 (±1.91)	5 (±1.41)	0.28
Number of lesions, n			1.0
Single	17	1	
Multiple	25	1	
Intravesical Bacillus Calmette-Guérin (BCG) therapy, n			0.44
Yes	10	1	
No	32	1	

## Discussion

An accurate histopathologic grading remains an important step in the management of noninvasive urothelial neoplasms because grading is a high-priority parameter for both prognostication and guidance of therapy in the urothelial group of neoplasms [9]. Therefore, pathologists and urologists have made several significant attempts in obtaining an optimal, effective, and reproducible grading system for papillary urothelial neoplasms [3,4,7].

In 2016, the WHO/ISUP classification system described the architectural and cytological features of all noninvasive papillary urothelial neoplasm in detail [5]. LG-UrCa is characterised by complex papillary architecture with an orderly arrangement of the cellular architecture with mild to minimal variability of the cytological features. In contrast, HG-UrCa is characterised by marked variability of the cellular architecture and cytological features appreciable at low magnification [5].

In the present retrospective study, we found that there was a very low rate of misclassification of HG-UrCa (4.16%; two out of 48 cases), which was much lower than reported by Miyamoto et al., whose rate of misclassification (under-grading of HG-UrCa) was 14.5% [10]. The low rate of misclassification may be attributed to the fact that we cater to a fair number of cases in our institute.

Herr et al. conducted a large cohort study evaluating 172 patients with LG-UrCa of the bladder and found a 72% recurrence rate and 10% progression rate in either grade (4%) or stage (6%). In contrast, other studies have demonstrated a wider range, with a recurrence rate of 34-72% and a progression rate of 4-10% [11-15]. Miyamoto et al. also found a recurrence rate of 53% and a progression rate of 18.3% [10]. In the present study, we found a recurrence rate of 30.4%, which was a little lower than previous studies, and our grade progression rate of 8.7% was similar to Herr et al. and other previous studies but lower than Miyamoto et al. [10,11]. The stage progression in the present study was 4.34%, which was lower than Herr et al. and Miyamoto et al. [10,11]. None of our patients died of urothelial carcinoma; however, one of the patients developed metastatic disease but was alive at the last follow-up.

Although the age of the patients had no significant association with tumour recurrence, grade, or stage, the mean age was higher for patients with tumour recurrence, grade, or stage progression. These findings are in line with Miyamoto et al. [10].

Tumour multiplicity at the initial presentation was the most predictive factor of recurrence, as shown in many previous studies [9,12,13,16]. In Herr et al., the recurrence rate was 39% in patients with a single lesion at initial diagnosis while an 85% recurrence rate was found in patients with multiple lesions at initial diagnosis [11]. In the present study, we found an 85.7% recurrence rate in patients with multiple lesions at initial diagnosis, which was statistically significant (p=0.02) and were comparable with many previous studies [9,12,13,16].

Intravesical BCG therapy remains an effective intravesical treatment for LG-UrCa. Since its introduction in 1976, several studies have shown that intravesical BCG therapy following transurethral resection of the bladder tumour (TURBT) is superior to TURBT alone as well as to TURBT plus intravesical chemotherapy [17]. Although the exact mechanism of action of BCG is not fully deciphered, it initiates a complex immunological cascade leading to a vigorous cellular immune response [17]. Although in the present study the intravesical BCG therapy showed no statistical difference (p=0.065) in patients with regard to recurrence, Millán-Rodríguez et al. have found that intravesical therapy with BCG decreased the risk of tumour recurrence [18]. This discrepancy in findings may be explained by the fact that only 11 patients (23.9%) had received intravesical BCG therapy in the present study.

We found that patients with larger tumour sizes at presentation had a higher rate of recurrence (p=0.009). This finding concurs with the studies done by Herr et al. and Millán-Rodríguez et al., who showed that tumours with larger size had a higher recurrence rate [9,18]. Although the tumour size of the patients at presentation had no significant association with tumour grade and stage, the mean initial tumour size was higher in patients with tumour grade and stage progression. These findings are similar to that of Miyamoto et al. [10].

In the present study, none of the clinicopathological parameters including tumour multiplicity, intravesical BCG therapy, or initial tumour size had any significant association with the tumour grade or stage progression, which was comparable to many prior studies [9-16]. Also, the number of patients with grade and/or stage progression was way too low to have any significant finding.

## Conclusions

The tumour recurrence in LG-UrCa patients was lower in our study as compared to other studies. However, these patients had progression to higher tumour grade, which was comparable with other studies. A small subset of patients, who had higher tumour grade progression, also showed higher TNM stage progression and eventually underwent radical cystectomy. Patients with larger tumour size and tumour multiplicity at initial presentation had a higher tumour recurrence rate.
